# Determinants of Preference and Consumption of Healthy Food in Children

**DOI:** 10.3390/foods11020203

**Published:** 2022-01-12

**Authors:** Monica Laureati

**Affiliations:** Department of Food, Environmental and Nutritional Sciences (DeFENS), University of Milan, Via Celoria 2, 20133 Milano, Italy; monica.laureati@unimi.it; Tel.: +39-(0)-250-319-188

According to recent findings, action is urgently needed to promote healthy eating habits among children, especially to increase daily consumption of fresh fruits and vegetables [1]. Ensuring healthy diet consumption is important not only for decreasing risks of noncommunicable diseases (NCDs) and improving general health later in adulthood, but also for achieving the UN Sustainable Development Goals (SDGs) related to no hunger, good health and well-being, quality education, no poverty and economic growth [2].

In recent decades, changes in dietary patterns and physical activity have been identified as likely contributors to rising childhood obesity [2]. Many countries of the WHO European Region are facing the so-called double burden of malnutrition, characterized by the simultaneous manifestation of undernutrition (stunting and wasting) or micronutrient malnutrition, along with overweight and obesity [3]. Helping countries to promote healthy nutrition as an effective measure to reduce NCDs is one of the priorities of the European Programme of Work 2020–2025 [1].

Food preferences, particularly in children, play a pivotal role in the prediction of human food choices and, thus, potentially in the development of overweight and obesity. Although seemingly simple, food choices represent one of the most complex behaviors in life and are the result of many interacting factors, including biological and physiological (e.g., genetics, sensory acuity, age, gender), psychological (e.g., behavioral traits) and environmental factors (e.g., situational and socio-cultural factors) as well as intrinsic (appearance, smell, taste and somatosensory characteristics) and extrinsic (e.g., claims and food labels) food properties [4]. A better understanding of the factors contributing to shape food preferences and choice from early age can be beneficial to tackle unhealthy eating habits and to prevent NCDs. The articles included in the present Special Issue show important and interesting advances and new approaches in this field.

Investigating children’s food preferences is important but requires age-appropriate and engaging tools, including hedonic as well as emotion measurements which have been shown to provide additional information beyond liking [5]. Sick et al. [5] verified the suitability of emojis to describe food experiences in children aged 9–13 years and concluded that emojis are correctly discriminated along their valence (positive vs. negative) and power (dominant vs. submissive) dimension, and to a lower extent along the arousal dimension (high vs. low activation), indicating that this tool can be used in innovative methodological approaches that can help to better understand how pre-adolescents perceive and like sensory properties of food.

Sensory properties are the main drivers of food preference and rejection, especially in children. Previous studies have identified appearance and texture as the main contributors to food acceptance in young consumers. Appealing presentation of meals as well as soft and easy texture are often reported to influence positively liking [6].

From a psychological point of view, food neophobia is considered one of the strongest predictors of the number of foods liked and tried by school-age children. It has also been associated with decreased dietary variety and a less varied range of food preferences, especially with regard to healthy foods. Food neophobia, defined as the rejection of new and unfamiliar food, is considered as a developmentally appropriate response against the ingestion of new and potentially toxic foods. The trajectory of this behavior is not clear but food neophobia has been reported to peak between 2 and 6 years of age; however, for some subjects it is a more persistent trait [6]. Previous findings have suggested a positive association between food neophobia and responsiveness to ‘warning’ food stimuli in adults [7]. This can in part explain the rejection of some types of plant-based food that naturally contains fibers and phytochemicals notoriously characterized by sour, bitter and astringent sensations. Although being food neophobia the main contributor to food rejections in children, little information is available on the association between this behavioral trait and individual differences in food responsiveness in children. Three papers published in the present Special Issue made interesting contributions to this topic. Appiani et al. [8] verified the cognitive and perceptive suitability of Von Frey filaments and a gratings orientation test in children of different ages (6–13 years) and compared lingual tactile sensitivity between children and adults using these tools. There were no differences in lingual tactile sensitivity between children and adults, nor between children of different ages. Although neither texture preferences nor food consumption were correlated with lingual tactile sensitivity, there was a weak but significant positive correlation between lingual tactile sensitivity to the finest Von Frey filament and food neophobia in the youngest age group (6–7 years), indicating that children with higher levels of food neophobia are more sensitive to oral tactile stimuli. These results were corroborated by Cappellotto and Olsen [9] who showed that children (6–13 years) preferring softer and non-particulate versions of foods were more neophobic and sensory sensitive across all sensory domains. Moreover, Sandvik et al. [6] in a cross-national study on the drivers of acceptance of high-fiber biscuits found that, overall, tempting appearance, sweet taste, chocolate flavor as well as a crunchy, smooth and soft texture contributed positively to liking, while visible dots, dry and sticky texture, and whole-wheat were negative contributors. Interestingly, they highlighted that, for children with high levels of food neophobia, appearance terms (visible lumps and whole-grain) were drivers of disliking, while cross-cultural differences in drivers of liking and disliking were particularly salient for texture attributes. Further research should explore if optimizing appearance and texture attributes could be a way to increase liking of healthy foods in neophobic children.

Taste perception is also important in predicting food choice in children. Although the association between taste sensitivity and food liking has been widely investigated over the decades, it is still uncertain how different taste sensitivity measures relate to each other and to food liking. Ervina et al. [10] explored the association between basic tastes, fattiness sensitivity and reported food liking in 11-year-old children using different methodological approaches (i.e., detection and recognition thresholds, taste responsiveness, PROP responsiveness and paired comparison test). They found that reported food liking poorly correlated with taste sensitivity measured in water solutions and was positively driven by sweetness and fattiness characteristics, while bitterness and umami contributed negatively. Stronger associations were found between reported liking for fatty food and fattiness sensitivity measured in milk, with non-fat sensitive children preferring high-fat milk samples and declaring higher liking for fatty foods. On one hand, these findings highlight the need to explore the association between taste perception and liking using real food to achieve more representative results and suggest that individual variation in fattiness perception could be a potential contributor to the selection of fatty foods. On the other hand, weak correlations found the in the study may depend on the fact that food liking in children could not be influenced by taste sensitivity alone but by other factors such as familiarity, family and cultural background as well as digital devices.

In the recent decades, the use of screen-media devices (e.g., television, videogames) has been reported to influence lifestyles and eating patterns in children and adolescents. Prolonged use of screen-media devices, exacerbated during the SARS-CoV2 pandemic, has been described as a significant contributor to poor eating habits in children and adolescents, including higher propensities to consume sweets and fatty foods, and reduced intake of fruits and vegetables, as well as to sedentary lifestyles determining the development of overweight and obesity. Sina et al. [11] evaluated the associations between different types of digital media including television, computer/game console and smartphone use, as well as the exposure to internet content and children’s and adolescents’ (age range 6–17 years) taste preferences for sweet, fatty, salty, and bitter, in a large sample of nearly 7000 individuals from 7 European countries. They found that European children and adolescents spent 2.4 h/day on average in front of screens, with 54.8% of them exceeding the WHO guidelines. Moreover, increasing exposures to digital media were associated positively with sweet, fatty and salty taste preferences, while the inverse was true of bitter preference, thus highlighting the need to implement effective strategies that involve families, pediatricians and policy makers to limit children’s and adolescents’ exposure to digital media content improving their eating habits and preventing childhood obesity-related comorbidities.

Other strategies that seem promising in guiding children toward healthy eating habits are school-based multicomponent interventions based on different principles, among which repeated exposure is reported to be the most effective in changing eating behavior. The mere-exposure effect is a phenomenon through which individuals tend to develop a preference for a stimulus merely because they are familiar with it. Novel stimuli initially elicit a reaction of fear and avoidance (i.e., the aforementioned food neophobia), which can be reduced by repeated exposure to them. The effectiveness of repeated exposure to reduce neophobic reactions and to promote the consumption of fruits and vegetables has been tested in several studies with mixed results [12]. Repeated exposure can be more or less effective depending on a myriad of factors including the number and frequency of exposure, type of food stimulus delivered, age of the children. In this Special Issue, four articles made interesting contributions within this research topic. Karagiannaki et al. published two papers to determine optimal exposure frequency [12] and stimulus shape [13] for introducing a novel vegetable among 3–6-year-old children. In a first study [12], they tested seven exposures delivered in kindergartens twice a week, once a week and once every second week. All exposure frequencies lead to an increase in liking and intake of daikon with a plateau reached at the fourth exposure for all the groups including the control group, suggesting that even low frequencies may provide positive changes in acceptance. In a second study [13], they tested intervention groups that were exposed seven times (twice per week) to daikon served in one of three different serving styles: sticks, triangles or grated. They concluded that mere exposure was efficient towards increasing liking and intake of the novel vegetable with all the shapes to deliver positive results, indicating that no particular serving style can be recommended.

Hildrey et al. [14] tested the suitability of an online survey technology with repeated exposure to tailored health promotion messaging in a school-setting. The approach, adapted from a clinical setting, was found to be acceptable and useful in assessing diet quality and physical activity among low-income students aged 10–14 years.

Johansson et al. [15] investigated if early repeated exposure to Nordic fruits and vegetables in a protein-reduced, Nordic complementary diet has long-term effects (>12 months) on eating behavior and food acceptance compared to a conventional diet for infants until 18 months of age. Parents were provided with a taste portion schedule for homemade repeated exposure, protein-reduced baby food products and baby food recipes with fruits and vegetables, as well as educational support through social media. Daily intake of fruits and vegetables at 12 months was higher in the Nordic diet group compared to the conventional diet group. From 12 to 18 months, fruit and vegetable intake decreased, but the Nordic diet group still consumed 32% more compared to the conventional diet group. Overall adherence to the Nordic diet was high, indicating that repeated exposure combined with education and support to parents may impact the child’s food intake with infants willing to consume foods characterized by unfamiliar flavors (bitter, sour and astringent) which are usually not present in conventional complementary feeding.

Parents play a fundamental role in the development of children’s food choices. Mothers shape their baby’s food preferences even prior to birth through the amniotic fluid and then postnatally through breastfeeding. With complementary feeding, parents begin to influence their child’s food choices also acting as models [16]. Estay et al. [17] explored cross-cultural variables involved in children’s vegetable consumption through the analysis of mothers’ perceptions, attitudes, and feeding practices regarding their children’s intake. Independently of cultural background, mothers agreed on the importance of children’s vegetable consumption, the influence that mothers have over their children’s vegetable intake, and how challenging it is to encourage children to eat a variety of vegetables. Ethnic groups differed regarding how they perceived the level of mothers’ responsibility over children’s vegetable intake, the way that mothers defined the amount of vegetables that children should eat, the constraints that mothers had on increasing their children’s vegetable intake and mothers’ recommendations to encourage vegetable consumption, suggesting that culture-specific strategies should be considered to foster healthy dietary habits in children.

Traditionally, mothers have played a greater role than fathers in food provision but a recent increase in fathers’ involvement in feeding their children has shifted the interest in studying the family as a whole [16]. In this context, Kähkönen et al. [16] found that, in a sample of 3–5 years old Finnish children, food preferences were more similar to the father’s rather than the mother’s preference. Moreover, Erhardt and Olsen [18] concluded that fathers can be more influential than mothers in reducing meat consumption in children as they found that fathers showed a higher meat attachment than mothers and that an increased meat attachment is associated with increased meat intake of the child, which elsewhere is accompanied by reduced consumption of vegetables.

In conclusion, the papers published in the present Special Issue contributed to the progress of sensory and consumer science with children and to shed light on the determinants of healthy eating in early life. Considering the complexity of the interacting factors underlying food preference and choice and the importance these behaviors have in healthy development of children, more research is definitely necessary in order to guide the consumers of the future toward healthy eating and lifestyles and to achieve more efficient and sustainable strategies that account for individual differences in food perception and personality traits and are based on family sensory and nutritional education.
